# Multifunctional Shape-Memory Polyurethane/MnO_2_ Composites for Postsurgical Osteosarcoma Adaptive Treatment

**DOI:** 10.3390/ma19081504

**Published:** 2026-04-09

**Authors:** Deju Gao, Yuhan Du, Junjie Deng, Zhengxin Gan, Wei Zhang, Yuxiao Lai, Yuanchi Zhang

**Affiliations:** 1 Centre for Translational Medicine Research and Development, Shenzhen Institutes of Advanced Technology, Chinese Academy of Sciences, Shenzhen 518055, China; 2 University of Chinese Academy of Sciences, Beijing 100433, China; 3 Key Laboratory of Biomedical Imaging Science and System, Chinese Academy of Sciences, Shenzhen 518055, China; 4 Guangdong Engineering Laboratory of Biomaterials Additive Manufacturing, Shenzhen 518055, China

**Keywords:** shape-memory polyurethane, MnO_2_, adaptive, antitumor effects, bone regeneration

## Abstract

Treatment of postsurgical osteosarcoma remains one of the major challenges in orthopedic clinics. Conventional implants often fail to address complex pathological issues, including irregular bone defects, residual tumor cells, and delayed bone regeneration. Herein, this study reports a multifunctional shape-memory polyurethane (SMPU)/manganese dioxide (MnO_2_) composite that provides adaptive support, antitumor activity, and osteogenic bioactivity. SMPU was synthesized by introducing 1,4-butanediol (BDO) and dimethylolpropionic acid (DMPA) as chain extenders at a specific ratio. Commercial MnO_2_ nanoparticles were incorporated as both a photothermal agent and a bioactive component to achieve multifunctionality. As designed, a coordination system was formed between the polymer chains and MnO_2_ nanoparticles within the composites. The influence of MnO_2_ content was systematically investigated. Although increasing MnO_2_ amounts improved photothermal and mechanical performance, excessive incorporation adversely affected the molecular structure and compromised the composite’s biocompatibility. By adjusting the MnO_2_ content, the composites were demonstrated to possess robust mechanical performance, good shape-memory behavior, and controllable Mn^2+^ release. Additionally, the composites exhibited tunable photothermal performance under near-infrared (NIR) irradiation. Furthermore, in vitro studies confirmed that the composites containing 4 wt% MnO_2_ could eliminate tumor cells via photothermal effects and promote the osteogenic differentiation of human bone marrow-derived mesenchymal stem cells (hBMSCs). Overall, the SMPU/MnO_2_ composites had superior multifunction for treating irregular bone defects following bone tumor surgery.

## 1. Introduction

Osteosarcoma (OS) is one of the most common bone malignant tumors, primarily affecting adolescents and young adults [1,2]. Clinicians typically managed OS through surgical resection combined with chemotherapy or radiotherapy [3]. However, the persistence of occult residual tumor cells within the bone defect site may simultaneously impair bone regeneration and increase the risk of local recurrence [4,5]. Meanwhile, conventional postoperative chemotherapy or radiotherapy is limited by nonspecific drug distribution and systemic toxicity, which may also be insufficient to eradicate residual tumor cells locally and can further perturb host immune and reparative responses [6,7]. Therefore, treatments for bone reconstruction after OS surgery using biomaterials implants have gained increased attention [8,9,10]. Due to pathological characteristics of OS-induced bone defects, the biomaterials are expected to not only provide mechanical support for large defects but also deliver coordinated local therapy to promote osteogenesis while suppressing recurrence. In particular, the invasive progression of OS and the necessity for wide resection often create large, geometrically irregular defects [11,12]. As a result, the implanted biomaterials cannot adequately fill within the defect site. This predisposes the materials–tissue interface to micromotion and stress concentration under physiological loading, thereby compromising subsequent integration and regeneration [13,14,15]. It has been previously reported that some scaffolds could ablate residual tumor cells through photothermal effects, and that injectable biomaterials could improve defect filling in irregular postoperative sites [16,17]. However, the photothermal scaffolds generally lack self-adaptability and bioactivity, while the injectable systems often suffer from insufficient mechanical support. Therefore, a multifunctional biomaterial capable of in situ self-adaptation, osteogenesis promotion and tumor-recurrence prevention is highly desirable to treat post-OS bone defects.

Stimuli-responsive polymers can alter their macro/microstructures and properties based on changes in external environment, which have become one of the most attractive self-adaptive implants in the biomedical field [14,18,19,20]. Among them, shape-memory polyurethane (SMPU) has exhibited superior structural adaptability and mechanical performance in the reconstruction of irregular bone defects [21,22]. Prior to implantation, SMPU can be compressed or molded into a temporary configuration, recovering its preprogrammed shape under thermal activation upon placement within the defect cavity, enabling in situ conformal adaptation and self-fixation within complex geometries [23,24]. For example, Yang et al. demonstrated that the adjustment of the formulation ratios during SMPU synthesis yielded materials that combined high mechanical strength with an appropriate elastic modulus, thereby providing sustained and stable mechanical support during the early stage of bone repair [25]. Our previous studies also proved that the SMPU-based implants could achieve significantly improved stiffness and bioactivity for bone regeneration [26]. Therefore, SMPU is a promising matrix material for repairing irregular bone defects due to its provision of strong structural conformability and mechanical support. On the other hand, structural support alone is still insufficient for addressing the biological challenges after OS resection, e.g., locally residual tumor cells and new tissue growth within the defect region [27,28]. Existing shape-memory scaffolds have made certain progress in the postoperative management of bone tumors. For example, researchers introduced some magnetic nanoparticles (e.g., MoS_2_, NbC, Fe_3_O_4_) to achieve magnetically responsive shape-memory or photothermal functions [29,30]. But their osteogenic bioactivity and responsiveness to the tumor microenvironment often remain limited. Manganese dioxide (MnO_2_) has been widely studied for biomedical applications in recent years due to its strong photothermal conversion capability and responsiveness to the tumor microenvironment (TME) as well as its good pro-osteogenesis functions [31,32]. Previous publications have shown that MnO_2_ can generate localized heating under near-infrared (NIR) irradiation, enabling mild photothermal therapy to suppress tumor cell activity [33,34]. Meanwhile, in an acidic and H_2_O_2_/glutathione (GSH)-rich TME, MnO_2_ can undergo decomposition with the release of Mn^2+^ that can adaptably treat the tumors through activating immune response [35,36,37,38]. In addition, MnO_2_ and released Mn^2+^ have been reported to participate in the regulation of bone metabolism and to promote osteogenesis-related cellular behaviors [39,40,41].

**Figure 1:**
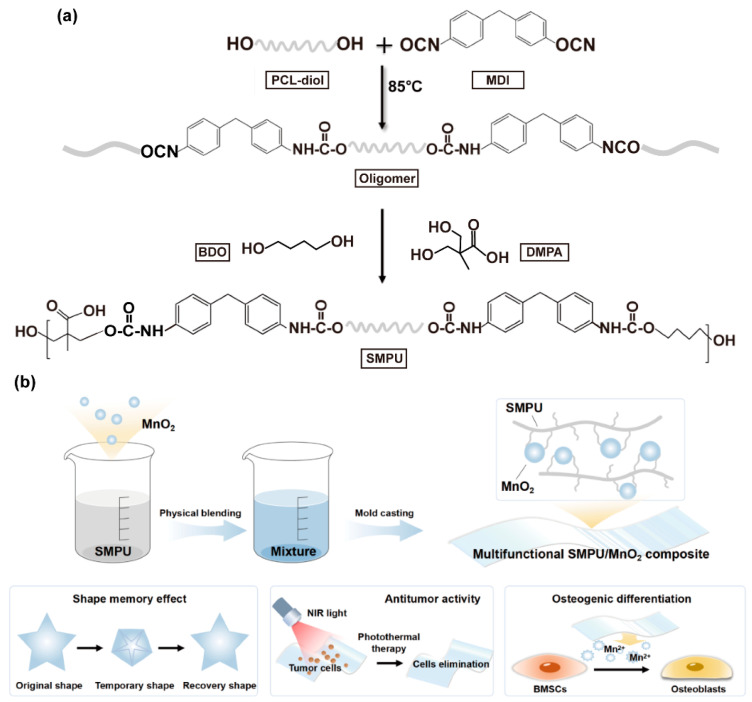
Schematic illustration of SMPU/MnO_2_ composites for post-OS adaptive treatment. (**a**) Synthesis of the SMPU. (**b**) Preparation of the composites and their multiple functions.

In this work, we developed a multifunctional bioactive composite by incorporating MnO_2_ into a poly(ε-caprolactone) (PCL)-based SMPU matrix for repairing irregular bone defects after OS surgery. SMPU was synthesized from PCL-diol, methylene diphenyl diisocyanate (MDI), 1,4-butanediol (BDO) and dimethylolpropionic acid (DMPA) (Figure 1a). Importantly, DMPA introduced carboxyl groups into the SMPU polymer chains, which served as chelation sites to promote homogeneous incorporation of MnO_2_ within the SMPU matrix, rather than simple physical blending. In addition, the composites could accelerate Mn^2+^ release under simulated tumor microenvironment conditions. After the addition of the MnO_2_, the composite was endowed with favorable self-adaptability, antitumor effects, and promotion of bone regeneration due to improved mechanical properties, photothermal effects, shape-memory functions, and bioactivities (Figure 1b). The structure and properties of the pristine SMPU with different component ratios were first characterized and compared. After obtaining optimized SMPU, various MnO_2_ nanoparticles were incorporated to prepare the composites. Next, the SMPU/MnO_2_ was comprehensively investigated in terms of its structure, mechanical and thermal properties, ions release, shape-memory effects, as well as in vitro biological performances relating to bone regeneration and tumor recurrence.

## 2. Experimental Section

### 2.1. Materials

PCL-diol (Mn ~4500, Sigma-Aldrich Co., Ltd., Shanghai, China), MDI, BDO, DMPA, N,N-dimethylacetamide (DMAC, HPLC grade), tetrahydrofuran (THF), glutathione (GSH), and hydrogen peroxide (H_2_O_2_) were purchased from Aladdin Biochemical Technology Co., Ltd. (Shanghai, China). Manganese dioxide (MnO_2_) nanoparticles (60–100 nm) were obtained from J&K Scientific, Beijing, China. The PCL-diol and BDO were dried prior to use under vacuum at 100 °C for 24 h to remove the moisture.

### 2.2. Preparation of SMPU and SMPU/MnO_2_ Composites

SMPU was synthesized via a prepolymer method using the BDO and/or DMPA as chain extenders. Briefly, PCL-diol and MDI were first reacted in an oil bath at 85 °C under vacuum to form an isocyanate-terminated prepolymer, with the DMAC solvent employed to adjust the viscosity. Subsequently, BDO/DMPA solutions with different ratios (1:0, 0.75:0.25, 0.5:0.5, 0.25:0.75, and 0:1) were added into the prepolymer system for chain extension. After reaction for certain period, the mixture was cast into a polytetrafluoroethylene mold and cured at 80 °C for 16 h to obtain the SMPU product (denoted as S1, S2, S3, S4, S5 according to the BDO/DMPA ratio). For bioactive composites, MnO_2_ nanoparticles with different contents (2 wt%, 4 wt%, 6 wt%, and 8 wt%, relative to the SMPU weight) were introduced into the selected SMPU solution, followed by mechanical stirring and ultrasonication to achieve uniform dispersion. The suspensions were then cast via solvent evaporation to form various SMPU/MnO_2_ composites (denoted as SMn2, SMn4, SMn6, SMn8 according to the MnO_2_ content).

### 2.3. Characterization of SMPU and SMPU/MnO_2_ Composites

The molecular weight and molecular weight distribution of various SMPU were determined using gel permeation chromatography (GPC, Agilent 1260, Waldbronn, Germany). The chemical structures of SMPU and SMPU composites were investigated using a Fourier transform infrared spectroscopy (Frontier, Perkin-Elmer, Waltham, MA, USA) and an X-ray diffractometer (D8 Advance, Bruker, Karlsruhe, Germany), respectively. The morphologies of the SMPU and its composites were explored by a field-emission scanning electron microscope (FE-SEM, ZEISS Sigma 300, Carl Zeiss, Oberkochen, Germany) combined with an energy-dispersive X-ray spectroscopy (EDS, XFlash 6130, Bruker, Germany) at an accelerating voltage of 15 kV with a working distance of approximately 8.5 mm. Surface wettability was examined by the standard water contact angle goniometer (Dataphysics, OCA, 15EC, Filderstadt, Germany). ~4 µL deionized (DI) water was dropped on the surface of SMPU and SMPU/MnO_2_ composites followed by data recording for further comparison. Thermal properties of the samples were evaluated by differential scanning calorimetry (DSC, NETZSCH DSC404F3 Pegasus, Selb, Germany) to obtain the melting temperatures (T_m_). The temperature range was −20 to 120 °C with a temperature rate of 10 °C min^−1^. The mechanical tests were conducted using an electronic universal testing machine (Shenzhen Wance Testing Machine Co., Ltd., Shenzhen, China) at a testing rate of 10 mm min^−1^ at room temperature, followed by calculating the modulus and stress from the stress–strain curves. The photothermal effects of materials were evaluated through a near-infrared (NIR) laser irradiation system (808 nm, P = 1 W cm^−2^, LWIRPD-10F, Laserwave, Beijing, China). The thermal images and corresponding data of temperature changes were recorded by a NIR camera (FLIR One, FLIR Systems, Inc., Hong Kong, China).

### 2.4. Shape-Memory Properties of the SMPU and Its Composites

The shape-memory properties were investigated through recording changes in the length at various conditions. The samples were first cut into the same size, and the initial length was recorded as L_0_. Then, the specimens were heated to ~60 °C for 5 min and stretched along the length direction to 100% strain (length recorded as L_1_). After cooling down to room temperature, the specimens were held for another 5 min to fix the temporary shape. The fixed length was recorded as L_2_. The specimens were subsequently reheated to 55 °C to trigger shape recovery. After recovery was completed, the specimen length was measured and recorded as L_3_. The shape-fixity ratio (R_f_) and shape-recovery ratio (R_r_) was calculated using the equations [26]:

(1) $$R_{f}=\frac{L_{0}-L_{2}}{L_{0}-L_{1}}*100\%$$

(2) $$R_{r}=\frac{L_{0}-L_{3}}{L_{0}-L_{1}}*100\%$$

where R_f_ stands for the capacity of the shape-memory composites to maintain their temporary shape. R_r_ represents the ability of the composites to recover their initial permanent shape. R_f_ and R_r_ are the two most important parameters of shape-memory properties.

### 2.5. In Vitro Degradation

Based on our previous study, the SMPU and its composites were used to evaluate degradative behavior and Mn^2+^ releasing properties [41]. The samples (initial weight recorded as *M*_0_) were immersed in the phosphate-buffered saline (PBS) buffer (pH = 7.4, 0.1 g mL^−1^) at 37 °C and 60 rpm. At predetermined time points (1, 3, 5, 14, 21, and 28 days), the samples were dried and then weighed, and the weight was recorded as M_t_. The weight loss ratio during degradation could be calculated according to equation:

(3) $$Weight\;loss\;ratio=\frac{M_{0}-M_{t}}{M_{0}}*100\%$$

In addition, the concentrations of Mn^2+^ released during degradation were quantitatively determined by inductively coupled plasma optical emission spectroscopy (ICP, Agilent 710, Santa Clara, CA, USA). To further simulate the TME, GSH (10 mM) and H_2_O_2_ (10 mM) solution were added in the PBS to prepare the TME degradation liquids. Then, the responsive released Mn^2+^ of the SMPU/MnO_2_ were measured using above methods.

### 2.6. In Vitro Experiments

In vitro cell studies were conducted to investigate the biological functions of the SMPU and its composites on promoting bone growth and preventing tumor recurrence. At first, all samples were sterilized by immersion in 75% (*v*/*v*) ethanol for 30 min, followed by ultraviolet (UV) irradiation on each side for 2 h. This method did not significantly affect the properties of the materials. The sterilized materials were immersed in the α-minimum essential medium (α-MEM, Gibco, Grand Island, NY, USA) supplemented with 10% (*v*/*v*) fetal bovine serum (FBS, Gibco, USA) and 1% (*v*/*v*) penicillin/streptomycin (Gibco, USA) at a defined ratio (0.1 g mL^−1^) for 24 h at 37 °C in a 5% CO_2_ atmosphere. Then, the human bone marrow mesenchymal stem cells (hBMSCs, passage number of 3–6, HUXMA-01001, Cyagen, Suzhou, China) were cultured using various extract medium in 24-well plates for designated periods, respectively. Cell viability was investigated through the live/dead staining methods after 1, 3, and 5 days. Alkaline phosphatase (ALP) staining assay was performed to evaluate osteogenic differentiation of the hBMSCs in various groups. In addition, sterilized SMPU/MnO_2_ composites were co-cultured with the human osteosarcoma cells (143B, CRL-8303, ATCC, Manassas, VA, USA) followed by being irradiated by NIR light (808 nm, P = 1 W cm^−2^) for 60 s. After further incubation for 2 h, cell viability was detected using the CCK-8 assay and live/dead staining to determine the photothermal antitumor function of the composites [42].

### 2.7. Statistical Analysis

Quantitative data were presented as the mean ± standard deviation (SD). Statistical analyses were carried out using the one-way ANOVA analysis by the software SPSS 27.0. The significant difference between the compared groups was set to *p* < 0.05. The graphics of the data analysis were prepared though the software Origin 2022.

## 3. Result and Discussion

### 3.1. Characterization of SMPU

**Figure 2:**
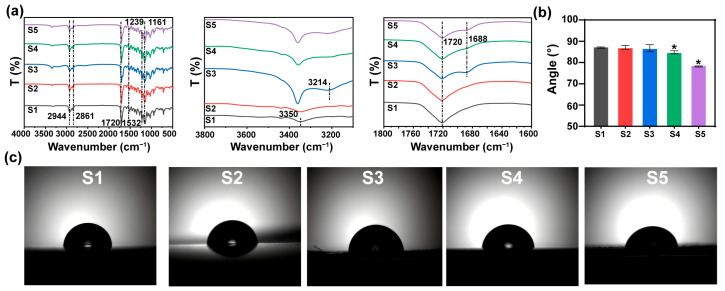
Characterization of SMPU. (**a**) FTIR spectra and the magnified view. (**b**) Quantitative water contact angles. (**c**) Images of contact angles on various SMPU surfaces. *, significant difference compared to S1, *p* < 0.05.

The molecular weights of SMPU with different DMPA ratios were analyzed by GPC (Table S1). All samples showed stable weight-average molecular weights (M_w_, 7–9 w) and similar dispersity values (PD~2.5), suggesting a stable approach and good reproducibility for synthesizing the SMPU [43]. FTIR results confirmed the successful synthesis of the SMPU (Figure 2a). All samples exhibited characteristic absorption bands, including N-H stretching at ~3350 cm^−1^, urethane-related C=O stretching at ~1720 cm^−1^, C-N stretching at ~1532 cm^−1^, -CH_2_ vibrations at ~2944 and ~2861 cm^−1^, and C-O stretching at ~1239 and ~1161 cm^−1^ [44,45]. In addition, the isocyanate (-NCO) bands near ~2270 cm^−1^ disappeared, indicating extensive -NCO consumption and successful polyurethane formation [46]. DMPA incorporation introduced additional carboxyl-related bands (e.g., bands around ~3214 cm^−1^ and ~1688 cm^−1^), which confirmed the presence of carboxyl functionalities and suggested potential interaction/coordination sites for MnO_2_ [47]. These enhanced interactions could contribute to the improved physical properties of the multifunctional composites. To assess surface suitability for cell biocompatibility, water contact angle measurement was conducted (Figure 2b,c). A notable decrease in contact angle was observed from ~87.8° of the S1 to ~78.7° of S5, proving the increased hydrophilicity after adding the DMPA [48]. This change was mainly attributed to the introduction of hydrophilic carboxyl groups from DMPA, which was consistent with the appearance of carboxyl-related absorption bands at approximately 3214 cm^−1^ and 1688 cm^−1^ in the FTIR spectra.

**Figure 3:**
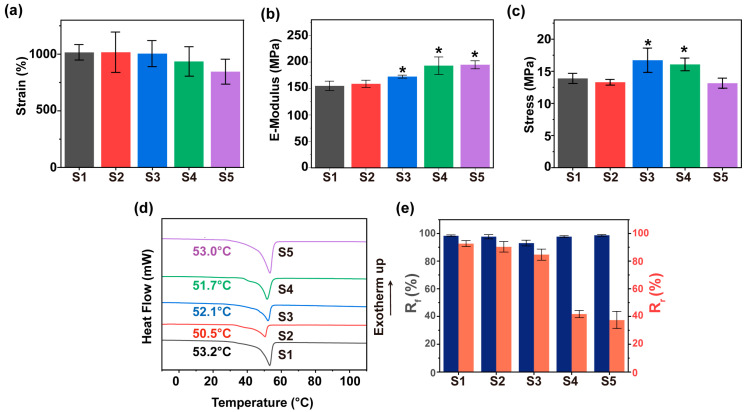
Physical properties of various SMPU. (**a**) Elongation at break. (**b**) Modulus. (**c**) Maximum tensile stress. (**d**) DSC results. (**e**) Shape-memory effects. *, significant difference compared to S1, *p* < 0.05.

To evaluate the impact of chain extender ratios, the mechanical properties of various SMPU samples were investigated. Specifically, the elongation at break of the samples decreased while their e-modulus increased as the introduced DMPA content augmented (Figure 3a,b). These could be ascribed to the introduction of carboxyl groups by DMPA, which enhanced intermolecular hydrogen bonding and restricted polymer chain mobility. In addition, the maximum tensile stress of the SMPU showed a trend of rising first and then falling (Figure 3c). The SMPU with a BDO:DMPA ratio of 0.5:0.5 (S3) achieved an optimal mechanical balance, exhibiting a peak strength of ~16.7 MPa. Given that osteosarcoma resection often results in irregular bone defects, the implant requires controllable shape-memory behavior to ensure adaptive conformability [26,49]. Therefore, thermal and shape-memory properties were subsequently evaluated. DSC results indicated the melting temperature (T_m_) of the SMPU fell into a biosafe range of 50–53 °C (Figure 3d). The S3 had the highest T_m_ among the hybrid series (S2–S4), suggesting a relatively ordered microstructure. Shape-memory tests confirmed that all samples maintained > 90% of R_f_, while the R_r_ declined with increasing DMPA contents (Figure 3e). Notably, the R_r_ values decreased sharply for S4 and S5, which could be attributed to the excessive carboxyl groups. The hydrogen-bonding networks formed by these groups impeded the segmental rearrangement required for shape recovery. Consequently, the S3 was selected for subsequent studies owing to its optimal mechanical balance (peak strength ~16.7 MPa) and the highest biocompatible T_m_ among the series. Furthermore, excess DMPA severely impaired shape recovery of the sample, whereas the S3 retained favorable overall shape-memory properties, rendering it suitable for fabricating shape-memory composites.

### 3.2. Structures and Morphologies of SMPU and Its Composites

**Figure 4:**
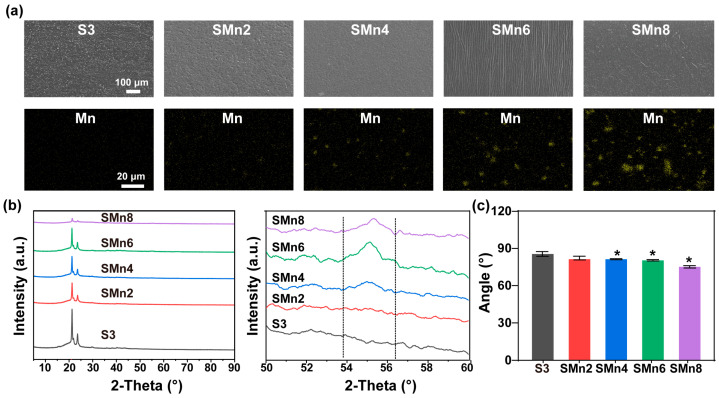
Characterization of SMPU and SMPU/MnO_2_ composites_._ (**a**) SEM-EDS images. Yellow: Element Mn. (**b**) XRD patterns and the magnified view. (**c**) Water contact angles. *, significant difference compared to S3, *p* < 0.05.

To further improve antitumor and osteogenic performance, various contents of MnO_2_ nanoparticles were incorporated into the S3 matrix to prepare the composites. As shown in Figure 4a, composites with different MnO_2_ contents displayed similar surface morphologies with moderate roughness, which is generally regarded as beneficial for cell adhesion and proliferation. In addition, the MnO_2_ nanoparticles were also homogeneously distributed inside the composites. XRD results confirmed the incorporation of the nanoparticles (Figure 4b). The crystalline peaks near 2θ = 20°corresponded to the reflection of (110) and (200) planes of PCL segments within the composites. Compared with the pristine S3, the diffraction intensities of the composites gradually decreased with increasing MnO_2_ contents, especially in the SMn8 group. This reduction might be caused by the steric hindrance and physical barrier effects of high-concentration MnO_2_ nanofillers, which disrupted the regular arrangement and microphase separation of the polyurethane chains, thereby inhibiting the crystallization of the soft segments. In the magnified view, the typical peaks of MnO_2_ appeared at 2θ = ~55° in the XRD patterns of the composites. Moreover, a shift in these MnO_2_-related peaks was observed with the MnO_2_ content increased, which could be attributed to the coordination effects between MnO_2_ and the SMPU matrix. Water contact angle measurements proved that the introduction of MnO_2_ nanoparticles further enhanced the hydrophilicity of the composites (Figure 4c).

### 3.3. Properties of the SMPU and Its Composites

**Figure 5:**
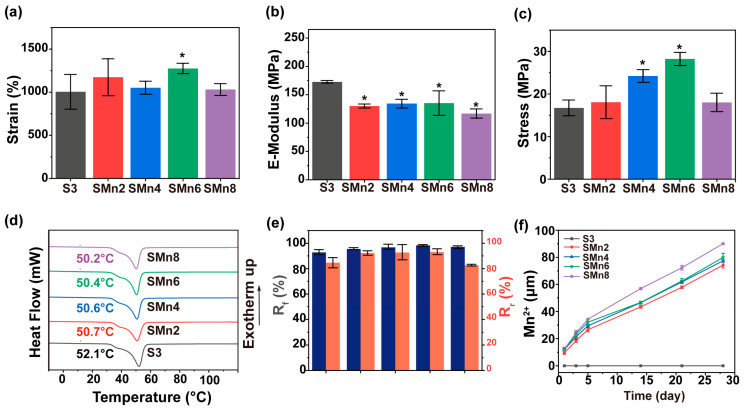
Physical properties of various SMPU and its composites. (**a**) Elongation at break. (**b**) Modulus. (**c**) Maximum tensile stress. (**d**) DSC results. (**e**) Shape-memory effects. (**f**) Cumulative release of Mn^2+^ within 28 days. *, significant difference compared to S3, *p* < 0.05.

The mechanical properties of the SMPU/MnO_2_ composite membranes were summarized in Figure 5a–c. Compared with the pristine S3, the composites showed higher elongation at break without an obvious rule (Figure 5a). Although the composites had a decreased modulus, their stiffness was enough to support the bone defects. For example, the modulus of the SMn4 and SMn6 was still more than 125 MPa (Figure 5b). In addition, the maximum tensile strength of the composites first increased and then decreased. Compared to that of the S3, the maximum tensile stress of SMn4 and SMn6 was respectively enhanced to ~25.2 MPa and ~28.2 MPa (Figure 5c). These results indicated a reinforcement effect at low-content loadings in the composites, whereas excessive fillers might cause crystallinity destruction of the polymer chains. DSC results demonstrated that the T_m_ of the composites decreased with increasing MnO_2_ content, which was likely attributed to destruction of the crystallization of the SMPU matrix (Figure 5d). Shape-memory tests proved that all composites had a good R_f_ of >95%. As for R_r_, SMn2, SMn4 and SMn6 had an improved performance (~92%, ~93%, ~93%, respectively), whereas SMn8 had only ~82% of R_r_. This might be caused by the impaired microphase separation process by excessive fillers during the shape recovery. Under simulated physiological conditions in vitro, the composites demonstrated negligible weight loss over 28 days, providing good mechanical support during early-stage repair (Figure S1). In addition, the SMPU matrix contained hydrolyzable segments, which endowed it with inherent biodegradability [50,51]. Therefore, the composite could degrade gradually under physiological conditions while maintaining structural stability for postsurgical antitumor intervention and facilitating ultimate bone regeneration. In addition, the composites could sustainably release Mn^2+^ under normal conditions (Figure 5f). Interestingly, the release of Mn^2+^ from the composites was significantly triggered under simulated TME conditions, where the cumulative release amount was 25 times higher than that under normal conditions (Figure S2). This TME-responsive release capability of the composites suggested the adaptive therapeutic potential for postsurgical osteosarcoma treatment.

### 3.4. Photothermal Performance and In Vitro Antitumor Effects

**Figure 6:**
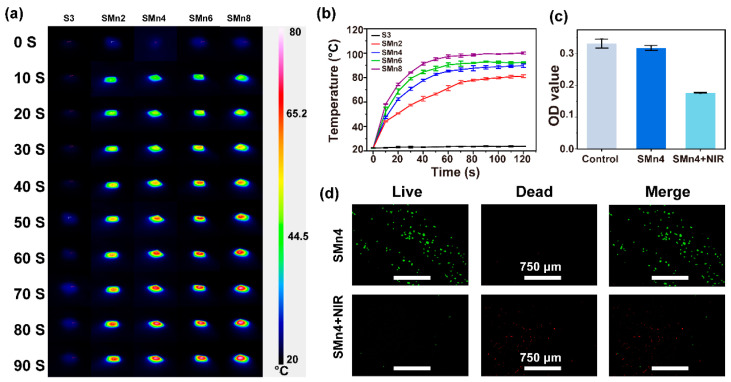
Photothermal performance and in vitro antitumor effects of SMPU and SMPU/MnO_2_ composites. (**a**) Infrared thermal images and (**b**) photothermal heating curves in a dry state under NIR irradiation (808 nm, 1 W·cm^−2^). (**c**) CCK-8 results and (**d**) live/dead staining images of 143B cells without and with NIR irradiation (808 nm,1 W cm^−2^) for 60 s. Green: live cells. Red: dead cells. Control group: cells cultured without samples or NIR irradiation. SMn4 group: cells cultured with SMn4 samples. SMn4 + NIR group: cells cultured with SMn4 samples under NIR irradiation.

To measure the photothermal performance, the heating profiles of composites with varying MnO_2_ contents were recorded under NIR irradiation (808 nm, P = 1 W cm^−2^) in a dry state (Figure 6a,b). The pristine S3 exhibited no thermal response to NIR irradiation, whereas MnO_2_-incorporated composites triggered a pronounced temperature rise. All MnO_2_-loaded groups rapidly reached ~43 °C within the initial 10 s. Notably, the SMn4, SMn6 and SMn8 groups could be heated to >80 °C within 60 s, suggesting good photothermal properties. Then, the photothermal therapy effects of the SMPU/MnO_2_ composites (SMn4) on the tumor cells were further evaluated (Figure 6c,d). In the absence of NIR irradiation, the 143B cells in the SMn4 group exhibited cell viability comparable to that in the control group. In contrast, after irradiating the composites with NIR for 60 s, significant cell death was observed in the SMn4 group. These findings underscore the potential of SMPU/MnO_2_ composites for application in photothermal tumor therapy.

### 3.5. In Vitro Cell Viability

**Figure 7:**
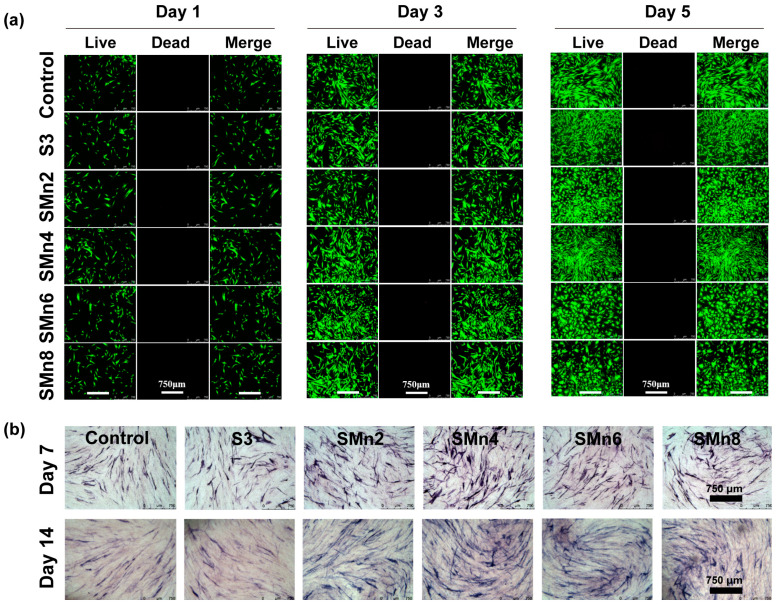
In vitro cell viability and osteogenic properties of SMPU and SMPU/MnO_2_ composites. (**a**) Fluorescence images of hBMSCs after incubation for 1, 3, and 5 days in various groups. Green: live cells. Red: dead cells. (**b**) ALP staining images of hBMSCs after incubation for 7 and 14 days in various groups. Control group: cells cultured without any samples.

The cytocompatibility and osteogenic properties of SMPU/MnO_2_ composites with varying MnO_2_ were further investigated. Live/dead staining results indicated that hBMSCs in all groups maintained good viability and proliferation capacity (Figure 7a). Notably, the SMn4 group showed superior cellular viability and relatively higher cell density compared with the other groups after 5 days of incubation. Nevertheless, cell proliferation in the SMn6 and SMn8 groups exhibited a slightly compromised trend with reduced cell density, implying that excessive Mn^2+^ might have negative effects on cell growth. In addition, hBMSCs cultured on SMn4 samples showed intact cytoskeletal structures after 48 h (Figure S3). These cells also indicated better alignment along surface microstructures compared with other groups, proving their robust adhesion to the composite surface. Regarding osteogenic properties, ALP staining results demonstrated the composites promoted osteogenic differentiation of the hBMSCs after incubation for 7 and 14 days, respectively (Figure 7b). Similarly, the SMn4 group had the most obvious improvement compared with other groups. In the SMn6 and SMn8 group, the ALP expression became less evident, suggesting the dosage effects on cell differentiation of Mn^2+^. At relatively high concentrations, Mn^2+^ might disrupt intracellular ion homeostasis and downregulate the expression of osteogenic-related genes, thereby inhibiting cell proliferation and osteogenic differentiation. Based on these results, SMn4 was selected as the optimal formulation, demonstrating a favorable balance between physicochemical properties and biological performance.

## 4. Conclusions

In this study, a multifunctional SMPU/MnO_2_ composite was designed and fabricated for adaptive bone repair, integrating shape-memory behavior, photothermal activity, and osteogenic bioactivity. The pristine SMPU with a BDO:DMPA ratio of 0.5:0.5 was first synthesized, which had balanced mechanical, thermal and shape-memory properties. Then, a series of SMPU/MnO_2_ composites were prepared through a physical blending method. The DMPA and MnO_2_ enabled the formation of the coordination system in the composites. Due to the dynamic molecular structure, the multifunctional composites had favorable mechanical, shape-memory, photothermal and biological properties. Specifically, the SMn4 composite had a modulus of a ~132.6 MPa and a maximum tensile stress of ~25.2 MPa. Moreover, the shape-fixity and recovery ratios of the SMn4 were more than 90%, which can provide adaptive support for the repair of the bone defects. In vitro degradation of the composites demonstrated the Mn^2+^ could sustainably release under normal physiological environment and rapidly release under the TME conditions. In addition, the composites had pronounced NIR responsive ability to achieve 80 °C within 60 s, which was proved to effectively kill the tumor cells. In vitro hBMSCs incubation experiments suggested the SMn4 composite had good cell viability and pro-osteogenic effects. Overall, the SMPU/MnO_2_ composites possessed multiple functions associated with antitumor activity and the promotion of bone regeneration, holding great promise for the treatment of post-osteosarcoma bone defects.

## Data Availability

The original contributions presented in this study are included in the article/Supplementary Material. Further inquiries can be directed to the corresponding authors.

## Supplementary Materials

The following supporting information can be downloaded at: https://www.mdpi.com/article/10.3390/ma19081504/s1, Figure S1: The weight loss of SMPU and SMPU/MnO_2_ composites during degradation for 28 days; Figure S2: Cumulative release of Mn^2+^ from the composite under normal conditions and tumor microenvironments within 28 days; Figure S3: Cytoskeleton staining images of the hBMSCs cultured on various samples after 48 h; Table S1: Molecular weights of SMPU with different BDO/DMPA ratios.
