# Supplementary material for: Multifunctional Shape-Memory Polyurethane/MnO2 Composites for Postsurgical Osteosarcoma Adaptive Treatment
Source: Materials (Basel). 2026 Apr 9;19(8):1504. doi: 10.3390/ma19081504 (PMC13117586; doi:10.3390/ma19081504)
Supplement: Supplementary file 1 [file materials-19-01504-s001.zip › materials-4219412-supplementary.pdf]

Supplementary Information

Multifunctional Shape-Memory Polyurethane/MnO_2 Composites for Postsurgical Osteosarcoma Adaptive Treatment

Deju Gao [1,2,†], Yuhan Du [1,2,†], Junjie Deng [1], Zhengxin Gan [3], Wei Zhang [1,2], Yuxiao Lai [1,2,3,4,*] and Yuanchi Zhang [1,2,*]

[1] Centre for Translational Medicine Research and Development, Shenzhen Institutes of Advanced Technology, Chinese Academy of Sciences, Shenzhen 518055, China
[2] University of Chinese Academy of Sciences, Beijing 100433, China
[3] Key Laboratory of Biomedical Imaging Science and System, Chinese Academy of Sciences, Shenzhen 518055, China
[4] Guangdong Engineering Laboratory of Biomaterials Additive Manufacturing, Shenzhen 518055, China
* Correspondence: yx.lai@siat.ac.cn (Y.L.); zhangyc@siat.ac.cn (Y.Z.)
† These authors contributed equally to this work.

Table S1. Molecular weights of SMPU with different BDO/DMPA ratios.

	Mp	Mn	Mw	Mv	PD
**S1**	83948	37180	87391	79221	2.3505
**S2**	75852	32444	82384	72914	2.5392
**S3**	82066	32678	84966	76368	2.6001
**S4**	80815	33046	84314	75681	2.5514
**S5**	65236	28438	71771	64357	2.5238

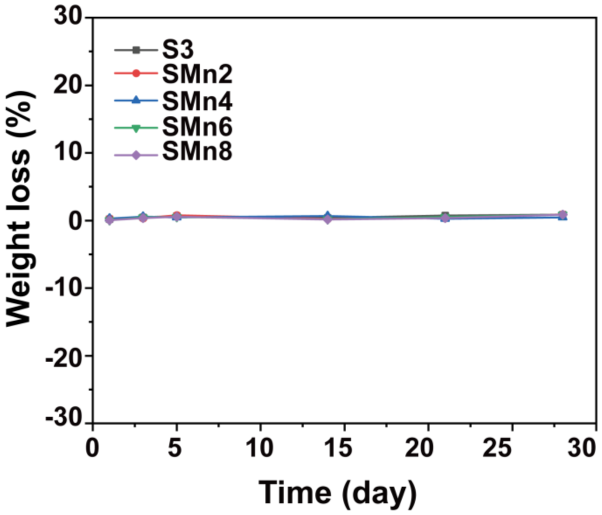

Figure S1. The weight loss of SMPU and SMPU/MnO_2 composites during degradation for 28 days.

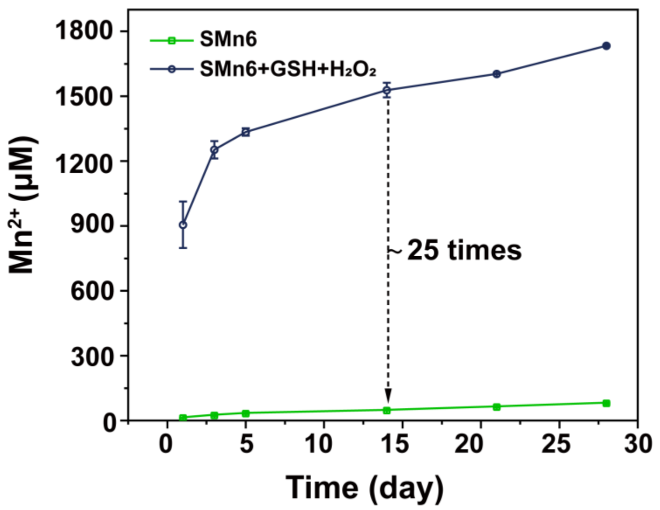

Figure S2. Cumulative release of Mn^{2+} from the composite under normal conditions and tumor microenvironments within 28 days.

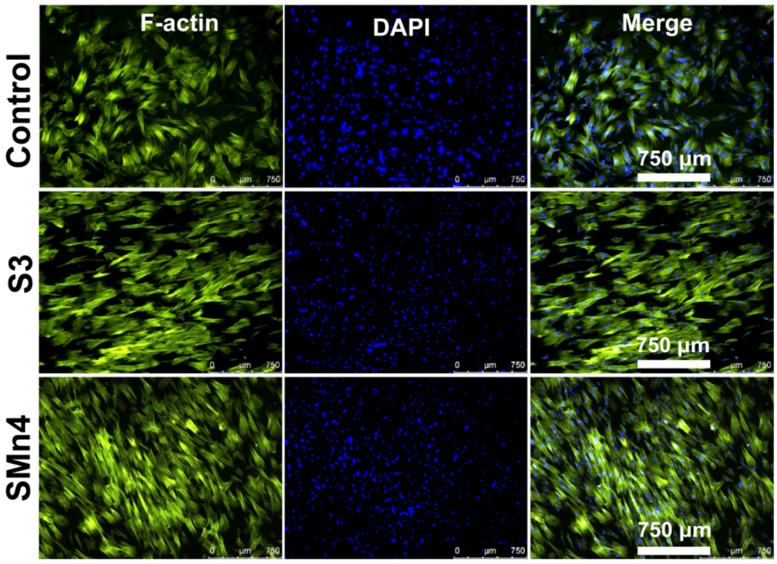

Figure S3. Cytoskeleton staining images of the hBMSCs cultured on various samples after 48 h.
